# Editorial: Genetics and epigenetics of psychiatric diseases—volume II

**DOI:** 10.3389/fgene.2022.1059855

**Published:** 2022-10-13

**Authors:** Qiyang Li, Yuanyuan Gai, Weihua Yue, Zhexing Wen, Cunyou Zhao

**Affiliations:** ^1^ Department of Rehabilitation, Zhujiang Hospital of Southern Medical University, Guangzhou, Guangdong, China; ^2^ Department of Medical Genetics, School of Basic Medical Sciences, Southern Medical University, Guangzhou, Guangdong, China; ^3^ Peking University Sixth Hospital & Institute of Mental Health, Beijing, China; ^4^ Department of Psychiatry and Behavioral Sciences, Emory University School of Medicine, Atlanta, GA, United States

**Keywords:** psychiatric disorders, genetics, epigenetics, environmental factors, molecule biomarkers, pathogenesis

Towards psychiatric disorders: Advance in understanding the role of mediating associations between genetic risk burden, environmental or epigenetic risk exposure and phenotype in psychiatric pathogenesis.

In this Research Topic, we have hosted 2 in-depth reviews and 10 original research articles introducing the potential mechanisms of how genetics, epigenetics and environmental factors interact and contribute to the etiology of psychiatric disorders.

Psychiatric disorders, including schizophrenia, bipolar disorder, major depressive disorder, and autism, are complex polygenic mental disorders which arise from their multifactorial nature: genetic, and epigenetic or environmental factors. Over the past decades, genome-wide association studies (GWASs) have reported a number of risk loci that are robustly associated with these disorders. These risk loci have not only provided pivotal insights into the genetic and biological bases of psychiatric disorders but also facilitated the translation of GWAS findings into potential therapeutics. Despite the great success of GWASs, GWAS loci are often hard to be interpreted: most common variants have a weak effect size on traits and exhibit combinatorial patterns of inheritance, thus probably conveying risks for the diseases through molecular networks and interactions. Furthermore, since the clinical presentations and severity of various subtypes of psychiatric disorders vary, genetic differences in various subtypes of psychiatric disorders remain unclear due to inadequate sample sizes or substandard clinical classifications. In addition, how environmental factors affect gene expression through epigenetic modifications and lead to psychiatric disorders remain unclear. Therefore, pinpointing the potential regulatory networks between genetic risk burden, environmental or epigenetic risk exposure and phenotype remains a challenge in psychiatric pathogenesis.

At the level of genomics, to find out the genetic differences in various subtypes of bipolar disorder, Huang et al. explored the potential causal associations between two bipolar disorder subtypes and lithium responses by comparing the difference in genetic structures among four different psychiatric traits with cross-phenotype analysis. This study illustrated genetic convergence and divergence between bipolar disorder I and II and provided new biological insights into psychiatric disorders. Given the important roles of *BDNF* and *CREB* in the development of schizophrenia, Ping et al. used a case-control design and discovered that rs11030101, rs2030324, rs6265, rs6740584 and rs2551640 are associated with schizophrenia. By using GWAS in Chinese students, Zhang et al. found out that rs80263879 and rs72478903 of *EPHX2* are candidate genetic loci for static spatial working memory. To identify hub genes in modules of major depressive disorder, Yang et al. used Weighted Gene Co-Expression Network Analysis and found six hub genes (*ADM*, *CITED2*, *IER5*, *NFKBIZ*, *SERTAD1*, *TNF*) with similar co-expression and dysregulation patterns in major depressive disorder. In addition, as *ULK4* is a rare susceptibility gene for psychiatric disorders, Luo et al. discussed the roles of ULK4 in neurodevelopmental and neuropsychiatric disorders which are helpful for the development of ULK4-based therapeutic strategy.

It the term of epigenetics, Zhang et al. conducted brain epitranscriptomic analysis in septic patients and identified that A-to-I RNA editing led to dysregulated gene expression, which eventually contributed to brain dysfunction. Given that RNA editing is understudied in the psychiatric field, this study thus provides valuable information to disease pathogenesis and open up a novel research direction. Moreover, Wang et al. identified 10 miRNAs which are dysregulated in Chinese autistic patients. As DNA methylation is an important epigenetic modification, Zhao et al. discovered that hypomethylation of *SSTR4* may contribute to the development of alcohol dependence by using BeadChip array and pyrosequencing. In addition, Jahangir et al. discussed the potentially etiological roles of long interspersed nuclear elements 1 in the development of schizophrenia.

Lipidomics has recently been developed as a powerful tool to investigate the natural characteristics of psychiatric disorders. Tao et al. suggested that peripheral blood lipidomic profile alterations could facilitate the identification of homogeneous transdiagnostic subtypes across psychiatric disorders which might help identifying patients with differential biological characterizations. Li et al. observed that *ERα* rs2234693 and rs9340799 polymorphisms are associated with susceptibility to major depressive disorder in women. The widely used atypical antipsychotics often associate with high prevalence of metabolic disorders in patients with schizophrenia. Yang et al. illustrated that mice with *Tcf7l2* deletion were more vulnerable to suffer metabolic abnormalities which may be mediated by GLP-1R after olanzapine administration.

Currently, the studies on the regulatory role of mediating associations between genetic risk burden, environmental or epigenetic risk exposure and phenotype in psychiatric disorders are still far from enough. Although it is premature to translate these newly found molecule biomarkers and pathogenesis into potential therapeutics, they do provide new insights into the etiology and treatment of psychiatric disorders that can ultimately guide clinical decision and therapeutics.

